# Protective Role of Peroxiredoxins against Reactive Oxygen Species in Neonatal Rat Testicular Gonocytes

**DOI:** 10.3390/antiox9010032

**Published:** 2019-12-30

**Authors:** Cristian O’Flaherty, Annie Boisvert, Gurpreet Manku, Martine Culty

**Affiliations:** 1 The Research Institute of the McGill University Health Centre, Montreal, QC H4A 3J1, Canada; cristian.oflaherty@mcgill.ca (C.O.); annieboisvert@hotmail.com (A.B.); gurpreet.manku@mail.mcgill.ca (G.M.); 2 Department of Surgery (Urology Division), McGill University, Montreal, QC H4A 3J1, Canada; 3 Department of Medicine, McGill University, Montreal, QC H4A 3J1, Canada; 4 Department of Pharmacology and Pharmaceutical Sciences, School of Pharmacy, University of Southern California School of Pharmacy, Los Angeles, CA 90089, USA

**Keywords:** testis, gonocytes, peroxiredoxins, oxidative stress, ROS, differentiation

## Abstract

Peroxiredoxins (PRDXs) are antioxidant enzymes that protect cells from oxidative stress and play a role in reactive oxygen species (ROS)-mediated signaling. We reported that PRDXs are critical for human fertility by maintaining sperm viability and regulating ROS levels during capacitation. Moreover, studies on Prdx6^−/−^ mice revealed the essential role of PRDX6 in the viability, motility, and fertility competence of spermatozoa. Although PRDXs are abundant in the testis and spermatozoa, their potential role at different phases of spermatogenesis and in perinatal germ cells is unknown. Here, we examined the expression and role of PRDXs in isolated rat neonatal gonocytes, the precursors of spermatogonia, including spermatogonial stem cells. Gene array, qPCR analyses showed that PRDX1, 2, 3, 5, and 6 transcripts are among the most abundant antioxidant genes in postnatal day (PND) 3 gonocytes, while immunofluorescence confirmed the expression of PRDX1, 2, and 6 proteins. The role of PRDXs in gonocyte viability was examined using PRDX inhibitors, revealing that the 2-Cys PRDXs and PRDX6 peroxidases activities are critical for gonocytes viability in basal condition, likely preventing an excessive accumulation of endogenous ROS in the cells. In contrast to its crucial role in spermatozoa, PRDX6 independent phospholipase A_2_ (iPLA_2_) activity was not critical in gonocytes in basal conditions. However, under conditions of H_2_O_2_-induced oxidative stress, all these enzymatic activities were critical to maintain gonocyte viability. The inhibition of PRDXs promoted a two-fold increase in lipid peroxidation and prevented gonocyte differentiation. These results suggest that ROS are produced in neonatal gonocytes, where they are maintained by PRDXs at levels that are non-toxic and permissive for cell differentiation. These findings show that PRDXs play a major role in the antioxidant machinery of gonocytes, to maintain cell viability and allow for differentiation.

## 1. Introduction

Peroxiredoxins are found in all living organisms, from bacteria, plants, yeasts to animals, where they act as scavengers of hydrogen peroxide (H_2_O_2_), lipid peroxides and peroxynitrite. Mammalian Peroxiredoxins (PRDXs) are important, not only as antioxidant enzymes preventing reactive oxygen species (ROS)-induced cell damage, but also as physiological regulators and sensors in a variety of cell and tissue types [1]. Indeed, the activation of several phosphatases, kinases, and tumor suppressor proteins have been shown to require a certain level of H_2_O_2_ acting as a second messenger in the vicinity of the enzymes, which is achieved by the transient and localized inhibition of PRDXs [2,3]. PRDXs are classified depending on the cysteine residues (Cys) in their active site, that will react with peroxides. They comprise the 2-Cys PRDX1 to 4, the atypical 2-Cys PRDX5, and the 1-Cys PRDX6, which has the particularity of being bifunctional, with both peroxidase and calcium-independent phospholipase A_2_ (iPLA_2_) activities [4]. The 2-Cys PRDX1 to 4 are homodimers in which the thiol of a cysteine residue of one PRDX subunit gets oxidized, then further reacts with the thiol group of the catalytic cysteine of the other subunit, forming a disulfide bond between the two subunits. By contrast, in the atypical PRDX5, 2 Cys of the same chain react upon oxidation to form an intrasubunit disulfide bond. Inactive PRDXs are then reactivated by a reduction of the disulfide bonds by thioredoxin (TRX), itself further reactivated by TRX reductase (TRD), using NADPH as a reducing equivalent. In the case of PRDX6, since the enzyme has only one catalytic Cys, the oxidized thiol will be reduced by the glutathione-GSH-transferase P1 (GSTP1) system [4,5,6]. 

Studies in PRDXs knockout mice have support the understanding of the diverse roles of these enzymes by highlighting the different defects in mice deficient for a specific PRDX [7]. In particular, spermatozoa have been shown to express the six PRDX isoforms [4], which act as ROS scavengers and are required to maintain viability as well as fertilizing competence [3,6,8,9,10]. While ROS are needed for sperm capacitation, due to their regulatory role in the phosphorylation of key proteins, their levels must be tightly controlled to prevent damaging oxidative stress, mainly by PRDX1 and 6 in rat [8,9,10]. In mice, PRDX6 deficiency or inhibition of its PLA_2_ activity were found to impair in vitro sperm fertilizing competence [11]. Low levels of PRDX6 were observed in infertile men, positioning PRDX6 as the first line of defense against oxidative stress in human spermatozoa [12]. Although spermatogenesis occurs in the testes of PRDX6 KO mice, these animals are subfertile, with defective and underperforming spermatozoa, suggesting potential alterations of some of the processes leading to sperm formation. While the importance of PRDXs on sperm integrity and function is clear, little is known on the role of PRDXs in germ cells from primordial germ cells to spermatids. 

The goal of this study was to examine the expression and role of PRDXs in neonatal gonocytes (also called pre-/pro-spermatogonia), the direct precursors of spermatogonial stem cells and first wave spermatogonia [13,14]. Gonocytes differentiate from primordial germ cells in the fetal gonad primordium, and undergo distinct phases of development, including successive phases of proliferation and quiescence in the fetus, resuming mitosis at postnatal day (PND) 3 in the rat, and simultaneously migrating toward the basement membrane of the seminiferous tubules where they differentiate to spermatogonia around PND6 [15]. We have previously shown that rat neonatal gonocyte differentiation is regulated by all trans-retinoic acid (RA) [16,17]. Extensive cell remodeling takes place during the proliferation, relocation and differentiation of neonatal gonocytes, in part regulated by the ubiquitin proteasome system [18]. We recently reported that neonatal gonocytes express high levels of cyclooxygenase 2 (COX2) and produce prostaglandins [19]. While COX2 and prostaglandins were reported to regulate ROS production in Sertoli cells [20], in other cell types such as the kidney mesanglial cell, ROS were shown to regulate COX2 expression and prostaglandin synthesis [21]. However, nothing is known on ROS formation and the antioxidant machinery in neonatal gonocytes. The present study demonstrates that PRDXs are essential for maintaining ROS homeostasis and cell viability in neonatal gonocytes, and that the iPLA_2_ activity of PRDX6 in these cells is not as critical as it is in spermatozoa, suggesting differential role for these antioxidant enzymes at different phases of germ cell development. 

## 2. Materials and Methods 

### 2.1. Chemicals

Conoidin A, an inhibitor of 2-cystein PRDX1-5 peroxidase activities was purchased from Cayman Chemical (Ann Arbor, MI, USA). MJ33 (1-Hexadecyl-3-(trifluoroethyl)-sn-glycero-2-phosphomethanol lithium), competitive inhibitor of the phospholipase A_2_ activity of PRDX6 was from Sigma-Aldrich (Milwaukee, WI, USA). Ezatiostat, a glutathione analog inhibitor of the Glutathione S-transferase P1 (GSTP1), required for the re-activation of the peroxidase activity of PRDX6, but not other PRDXs, was purchased from Sigma-Aldrich (Milwaukee, WI, USA). All-trans-retinoic acid, H_2_O_2_ and common reagents were from Sigma-Aldrich (Milwaukee, WI, USA).

### 2.2. Animals

PND2 newborn male Sprague Dawley rats were purchased from Charles Rivers Laboratories (Saint-Constant, Quebec, Canada). The pups were handled and euthanized according to the protocols approved by the McGill University Health Centre Animal Care Committee and the Canadian Council on Animal Care. USC Institutional Animal Care and Use Committee; Martine Culty protocol #20792-AM001 (Physiology and toxicology of male reproductive system).

### 2.3. Gonocyte Isolation and in Vitro Culture and Treatments 

Neonatal gonocytes were isolated by performing sequential enzymatic tissue dissociation together with mechanical dissociation of the pooled testes from 40 PND3 pups per experiment. This was followed by a step of differential overnight adhesion at 37 °C in medium containing 5% fetal bovine serum (FBS), and cell separation of the non-adherent cells on a 2–4% bovine serum albumin (BSA) gradient in serum-free medium on the next morning [22,23]. Enriched gonocyte preparations at 70–80% purity were obtained by pooling fractions containing high proportions of gonocytes, according to size and appearance, while a gonocyte purity above 95% was used for gene array analysis [18]. Freshly isolated gonocytes were cultured at 20 to 30,000 cells per well in 500 μL of RPMI 1640 containing 2.5% FBS, antibiotics, alone or with the PRDX inhibitors conoidin A, MJ33 and ezatiostat, and/or H_2_O_2_, at different concentrations, for 2 to 18 h, in 3.5% CO_2_, at 37 °C. Cell differentiation was examined by treating the gonocytes with 10^−6^ M retinoic acid (RA), in the absence or presence of the PRDX inhibitors. 

### 2.4. Cell Viability

Cell viability was assessed using a Trypan blue exclusion assay, by counting live and trypan blue-positive gonocytes as previously described [19], and viability was expressed as the mean ± SEM of the percentage of live cells against the total number of gonocytes in 3 independent experiments, each performed with triplicates.

### 2.5. RNA Extraction and Real-Time Quantitative PCR (Q-PCR) Analysis

Total RNA was extracted from cell pellets using the PicoPure RNA isolaton kit (Arcturus, Mountain View, CA, USA) and digested with DNase I (Qiagen, Santa Clarita, CA, USA), followed by cDNA synthesis with a single-strand cDNA transcriptor synthesis kit (Roche Diagnostics, Indianapolis, IN, USA), as previously described [18,19]. Quantitative real-time PCR (qPCR) was performed using SYBRgreen PCR Master Mix kit (Bio-Rad, Hercules, CA, USA) on a LightCycler 480 (LC480, Roche Diagnostics) [18]. The forward and reverse primers used are provided in Table 1. The comparative Ct method was used to calculate the relative expression of the differentiation marker Stra8, and PRDX 1 to 6, using 18S rRNA as housekeeping gene for data normalization. Changes in Stra8 gene expression are expressed as percent of the control values; and given as means ± SEM from 3 or 4 experiments, each using triplicates. PRDX data are expressed in relative gene expression and shown as the means ± SEM from 3 experiments. 

### 2.6. Gene Array Analysis

Briefly, the RNA samples isolated from three independent PND3 gonocytes and PND8 spermatogonia cell preparations, each made from multiple rat testes (each sample corresponding to the RNA of 60–90 pups for gonocytes, and 10 pup rats for spermatogonia) were analyzed using Illumina RatRef-12 Expression BeadChips, as previously described [18]. The relative levels of antioxidant genes were expressed in arbitrary units and presented as the means ± SEM of all data. 

As a comparison, the RNA from an enriched germ cell population prepared from the pooled testes of 4 PND60 adult rats was also analyzed in the gene arrays, as described before [24]. 

### 2.7. Lipid Peroxidation Measurement by Bodipy Labelling

Lipid peroxidation was measured using the fluorescent lipid peroxidation sensor BODIPY 581/591 C_11_, a reporter fatty acid labelled with bodipy (4,4-difluoro-3a,4adiaza-s-indacene) fluorophore, which can enter the cells and is used as a surrogate for endogenous lipids (Bodipy; Life Technologies (Burlington, Ontario, ON, Canada)). The peroxidation of the reporter fatty acid leads to a shift in the fluorescence of BODIPY from red to green in the cells. Thus, cells presenting green fluorescence corresponds to cells in which lipid peroxidation occurred, which can be quantified by assessing their proportion in each sample. Following 1.5 h of gonocyte treatments with either medium, PRDXs inhibitors and/or H_2_O_2_, the Bodipy reagent was added to the wells at 20 μM final concentration for an additional 30 min at 37 °C. The reactions were stopped by collecting and centrifuging the cells at 425× *g* for 10 min at 4 °C. The pellets were washed with PBS, the cells fixed with paraformaldehyde (3.5% final) for 7 min, washed and further collected by cytospin centrifugation on microscopic slides. For each slide, 5 pictures were taken using FITC (oxidized Bodipy reporter) and Texas Red (non-oxidized reporter) fluorescence, on a Leica fluorescent microscope. Lipid peroxidation was measured as the percent of Bodipy-positive green fluorescent cells over the total gonocyte numbers. Data are shown as the means ± SEM of samples from 3 different experiments. 

### 2.8. Immunohistochemistry (IHC)/Immunocytochemistry (ICC)

For IHC, PND3 testis samples were fixed with 4% paraformaldehyde solution (PFA), paraffin embedded, and 4 to 6 µm thick sections were made. IHC and ICC analyses were performed as previously described [18,19,23,25]. Briefly, the slides were dewaxed, rehydrated, treated for antigen retrieval, and then with a blocking reagent in PBS. The slides were then incubated overnight at 4 °C in Anti PRDX6 primary antibody diluted (1:100) in PBS containing 0.1% Triton X-100 and serum. This was followed by washes and 1-h incubation with a biotinylated secondary antibody (dilution 1:100) in 0.1% Triton X-100 and 10% BSA, at room temperature, then 15 min of treatment with Streptavidin-horse radish peroxidase and 15 min with an AEC Chromogen solution. Mayer’s hematoxylin was used for counterstaining, and Clear-Mount for coating. The slides were examined using bright-field microscopy. As negative controls, some slides were treated with non-specific immunoglobulin G instead of specific antibody. 

For ICC, the protein expression of PRDX1, 2, and 6 were examined in low purity gonocyte fractions pooled, washed with PBS, and fixed with 3.5% paraformaldehyde right after the BSA gradient. The fixed cells were collected by cytospin centrifugation, the slides dried and treated with acetone:methanol (60:40), followed by the antigen retrieval solution. The ICC reactions were similar to those described above for IHC, except for the use of fluorescent secondary antibodies. DAPI was used as a nuclear signal. Rabbit and mouse IgG were used as negative controls and gave no fluorescent signal (data not shown). Pictures of fluorescent signals and bright field were taken, using the same time of exposure for the specific antibodies and non-specific IgGs. Representative samples are shown. 

### 2.9. Statistical Analysis

Statistical analysis was performed using the unpaired Student’s t test or one-way ANOVA with post-hoc Tukey multiple comparison analysis, using Prism version 5.04 (GraphPad Software, San Diego, CA, USA). Changes with *p* values ≤ 0.05 were considered significant.

## 3. Results

### 3.1. PRDXs Are among the Highest Expressed Antioxidant Genes in Neonatal Gonocytes and in Spermatogonia

**Figure 1:**
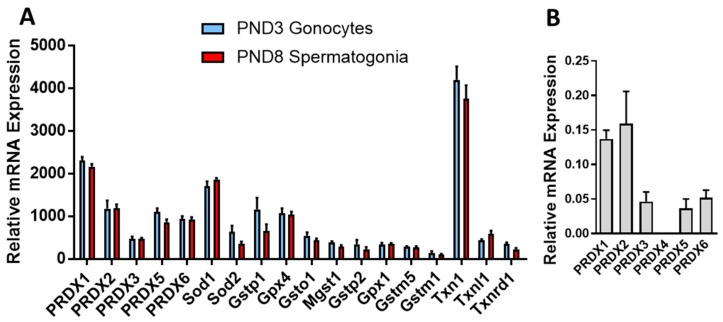
Relative expression of antioxidant genes in postnatal day 3 (PND3) gonocytes and PND8 spermatogonia. (**A**) gene array analysis of PND3 gonocytes and PND8 spermatogonia antioxidant genes. (**B**) qPCR analysis of PRDXs expression in PND3 gonocytes. Results represent the means ± SEM from 3 or 4 experiments, each using multiples animals.

Gene array analysis of antioxidant genes in highly purified rat PND3 gonocytes and PND8 spermatogonia showed that, at both ages, Prdxs were among the most abundant antioxidant genes (Figure 1A, Table S1). Comparing the signal intensities of all genes in the arrays showed that thioredoxin 1 (*Txn1*), *Prdx1* and superoxide dismutase (*Sod*) 1 were among the 1% most abundant genes in these cells, comprising relative signal intensities around and above 2000. Next, *Prdx2*, *Prdx5*, *Prdx6*, *Sod2*, *Gstp1*, and *Gpx4* were among the most abundant genes, with relative signal intensities from 600 to 1200 (Table S1). Other highly expressed genes (signal intensities between 300 and 600) included *Gsto1*, *Prdx3*, *Txnl1*, *Mgst1*, *Txnrd1*, *Gstp2*, *Gpx1*, and *Nrf2*. As a comparison, these genes had much higher signal intensities than 65 percent of the genes in the cells, which presented intensities below or around 20, levels found for Sod3 and Catalase. The ranking of expression for PRDXs were *Prdx1 > Prdx2 > Prdx5 > Prdx6 > Prdx3*, in both germ cell types. Measurement of the relative expression of the PRDXs by qPCR in PND3 gonocytes indicated a similar ranking of expression levels, where *Prdx1* and *Prdx2* were the most abundant, followed by *Prdx6*, *Prdx5* and *Prdx3*, and finally *Prdx4* present at very low levels (Figure 1B). The similarities in expression profiles of antioxidant genes between gonocytes and spermatogonia suggested a conserved antioxidant machinery between the two phases of germ cell development.

This was different from the levels found in enriched PND60 adult rat germ cells, in which the relative gene expression of *Gstm5* (6235) was the highest, followed by *Gpx4* (5507), *Hagh* (1141), *Sod1* (1136), *mGst1* (446), *Prdx1* (407), *Prdx6* (345), *Gstm1* (341), *Txn1* (335), *Prdx2* (223), *Prdx5* (204), *Gsto1* (188), *Prdx3* (144), *Gstt2* (140), *Txnl1* (132), *Txn2* (81), *Sod3* (73), *Gsta2* (64), *Gstp1* (65), and *Sod2* (44), the remaining genes being at very low expression levels.

**Figure 2:**
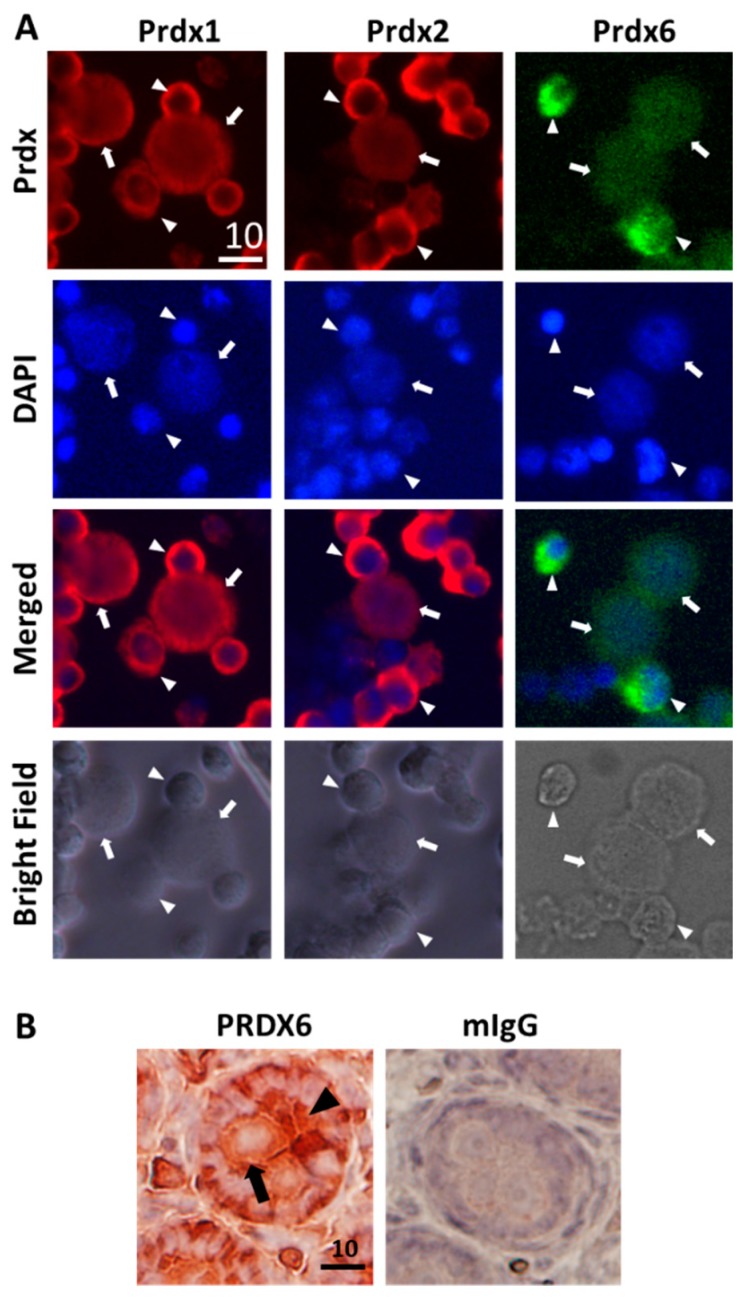
Protein expression of PRDX1, 2, and 6 in PND3 gonocytes. (**A**) Immunocytochemistry (ICC) analysis of PRDXs expression in cells collected right after the cell isolation procedure on cytospin slides. Low purity pooled fractions were used. White arrow: gonocytes. White arrowhead: somatic cells (Sertoli and peritubular myoid cells). (**B**) Immunohistochemistry (IHC) analysis of PND3 testis section. Representative pictures are shown. Black arrow: gonocytes. Black arrowhead: somatic cells. Scales are in μm.

The immunological analysis of the protein expression of PRDX1, 2, and 6 in mixed suspensions of PND3 gonocytes, Sertoli and myoid cells confirmed that the three PRDX proteins were expressed in gonocytes, Sertoli, and myoid cells, but at lower levels in gonocytes than in most of the somatic cells (Figure 2A). PRDX1 showed a slightly stronger signal than PRDX2, both being higher than PRDX6 in gonocytes, in agreement with the transcript levels. PRDX6 protein was also visible by IHC analysis in the cytoplasm of gonocytes in PND3 testis sections, but it was lower than the levels observed in Sertoli cells and some interstitial cells (Figure 2B). 

### 3.2. PRDXs Are Required for PND3 Gonocyte Survival in Basal Conditions

**Figure 3:**
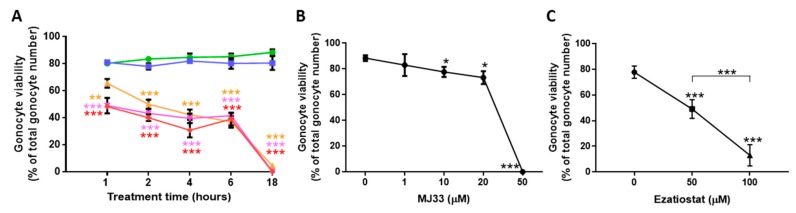
Effects of PRDX inhibitors on PND3 gonocyte viability. Cell viability was determined by trypan blue exclusion method. (**A**) Dose response and time course of conoidin A effects. Control: green; Conoidin A at 0.1 μM (blue); 1 μM (orange); 5 μM (pink); 10 μM (red). (**B**) MJ33 dose response at 18 h treatments. (**C**) Ezatiostat dose response at two hours of treatment. Results represent the means ± SEM from at least three experiments. Statistical significance: * *p* ≤ 0.05; ** *p* ≤ 0.01; *** *p* ≤ 0.001.

In view of the abundance of PRDXs in gonocytes, and knowing the importance of these peroxidases for spermatozoa viability and function, we examined whether inhibiting PRDXs would have an impact on the survival of PND3 gonocytes. For that, we performed dose-response and time-course studies in basal conditions where only endogenous ROS may be formed, using either the inhibitor of 2-Cys PRDXs, Conoidin A; the inhibitor of GSTP1 (that promotes PRDX6 glutathione-dependent re-activation), Ezatiostat; or the inhibitor of the phospholipase A_2_ activity of PRDX6, MJ33. Conoidin A induced a rapid dose-dependent decrease in gonocyte viability with concentrations of 5–10 μM, inducing 50% cell death after one hour of treatment (Figure 3A). There were no surviving cells with conoidin A concentrations of 1–10 μM after 18 h of treatments. By contrast, after 18 h of treatment with MJ33, there was no or minimally detrimental effects on cell viability with concentration from 1 to 20 μM (Figure 3B). However, at 50 μM, MJ33 did induce cell death after 18 h. Eztiostat dose responses were performed for two hours of treatment, because preliminary studies showed that longer time-periods induced the loss of a large amount of cells. Eztiostat inhibited cell survival in a dose-dependent manner (Figure 3C), with a concentration of 50 μM inducing nearly 50% of cell death. These data suggest that the peroxidase activity of 2-cys PRDXs and PRDX6 play a critical role in gonocyte protection against excessive endogenous ROS, and that inhibiting these enzymes even for a short time is sufficient to induce significant gonocyte cell death. Thus, PRDXs are required to maintain ROS homeostasis in neonatal gonocytes. By contrast, inhibiting the PLA2 activity of PRDX6 with MJ33 did not exert rapid deleterious effects on cell viability. 

**Figure 4:**
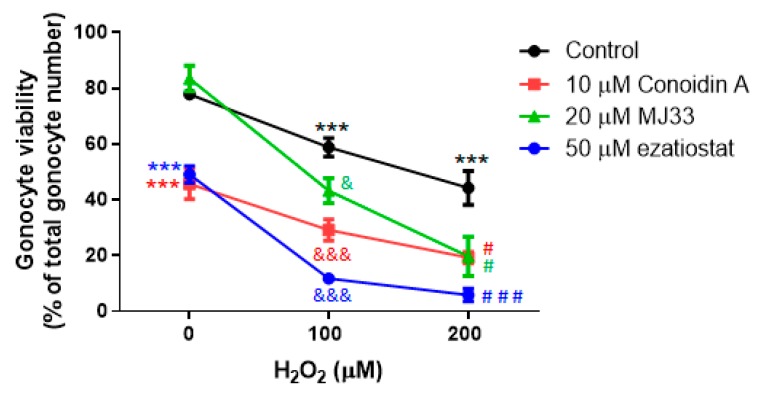
Effects of H_2_O_2_ and PRDX inhibitors on PND3 gonocyte viability. H_2_O_2_ and PRDX inhibitors were added to the cells at the indicated concentrations, alone or together for 2 h. Cell viability was determined by trypan blue exclusion method. Results represent the means ± SEM from at least three experiments. Statistical significance: *** *p* ≤ 0.001 treatment vs control; & *p* ≤ 0.05, &&& *p* ≤ 0.001 treatment vs 100 μM H_2_O_2_; # *p* ≤ 0.05, ### *p* ≤ 0.001 treatment vs 200 μM H_2_O_2._

PRDXs are required to prevent oxidative stress-induced cell death in PND3 gonocytes. Oxidative stress can be generated by external factors, as a result of exposure of the animals and their reproductive system to oxidative agents, or through the production of ROS by other testicular cell types in the vicinity of the gonocytes. Thus, we tested the ability of PRDXs to protect PND3 gonocytes from exogenous ROS, using H_2_O_2_ as an oxidative agent. The treatment of gonocytes with H_2_O_2_ at 100 and 200 μM for two hours induced a dose-dependent decrease in cell viability, with a 40% and 50% reduction in viability, respectively (Figure 4).

These damaging effects were significantly worsened by treating the cells with PRDXs inhibitors, with MJ33 having the mildest and ezatiostat the worst effects (Figure 4). These results indicate that the oxidative stress generated by H_2_O_2_ exerts an adverse effect on gonocyte viability, and that 2 Cys PRDX and PRDX6 peroxidase activities are required to protect gonocytes from oxidative stress. This further implies that other antioxidant proteins expressed in gonocytes, even those present at high levels such as Txn1, Sod1, and Sod2, are not able to rescue gonocytes from exposure to exogenous oxidants. Moreover, the fact that ezatiostat and MJ33 exacerbated the adverse effect of H_2_O_2_ on gonocyte viability suggests that both the peroxidase and phospholipase A_2_ activity of PRDX6 are important for gonocyte survival.

### 3.3. H_2_O_2_ Induces Lipid Peroxidation, and PRDXs Prevent Endogenous ROS-Induced Lipid Peroxidation in PND3 Gonocytes

**Figure 5:**
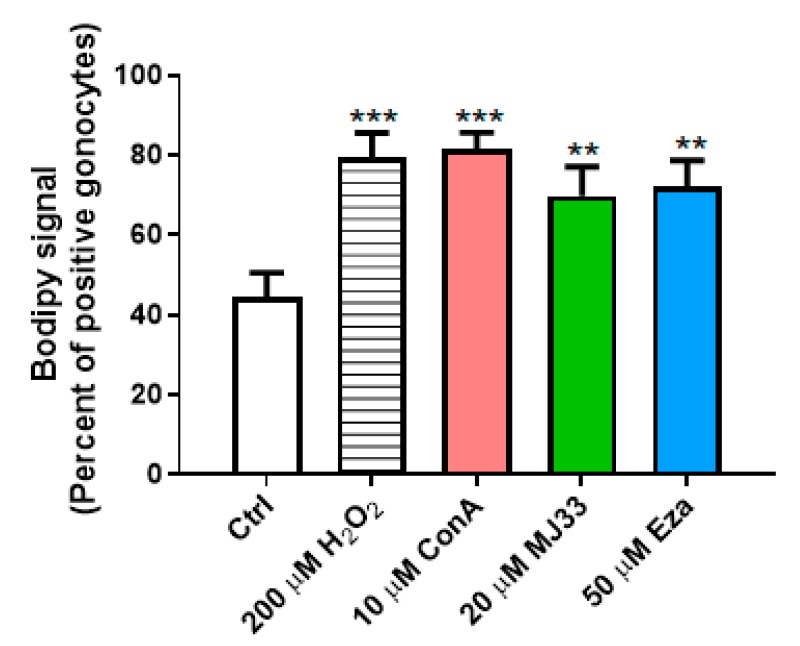
Effects of H_2_O_2_ and PRDX inhibitors on lipid peroxide formation in PND3 gonocytes. H_2_O_2_ and PRDX inhibitors were added for two hours on gonocytes. Lipid peroxidation was measured using the Bodipy assay. Results are the means ± SEM from at least three experiments. Statistical significance: ** *p* ≤ 0.01; *** *p* ≤ 0.001.

Lipid peroxidation has been known for decades to be one of the sources of spermatozoa damage in infertile men [26]. Thus, we examined whether lipid peroxidation could also play a role in the deleterious effects of H_2_O_2_ and PRDX inhibitors on gonocyte viability, using a fluorescent fatty acid reporter system that allowed quantifying the proportion of cells where lipid peroxidation had occurred. While lipid peroxidation was detectable in 40% of the control gonocytes, a condition corresponding to 80% cell viability, treatment with H_2_O_2_ (a condition decreasing cell viability by 50%) doubled the numbers of gonocytes presenting lipid peroxidation to nearly 80% of positive cells in gonocytes (Figure 5). To examine the effects of PRDX inhibitors, we used concentrations of conoidin A and ezatiostat decreasing viability by ~50% (10 and 50 μM respectively), and a concentration of MJ33 that did not significantly alter cell viability compared to controls (20 μM). Treatments with conoidin A alone had the same effect as H_2_O_2_ on lipid peroxidation, doubling the percent of gonocytes presenting lipid peroxidation, while MJ33 and ezatiostat induced significant but lower increases in lipid peroxidation, reaching 70% of positive cells, a 1.55 increase over the levels in control cells (Figure 5). 

Combining PRDX inhibitors with H_2_O_2_ did not further increase lipid peroxidation, which already affected most of the cells with individual treatments (data not shown). These results show that an oxidative stressor such as H_2_O_2_ induces similar levels of lipid peroxidation as endogenous ROS accumulating in the absence of PRDX activities in PND3 gonocytes. This suggests that lipid peroxides are produced physiologically in gonocytes, and that PRDXs are critical for preventing excessive levels that could affect gonocyte survival. The data also imply that PRDXs protect gonocytes from excessive endogenous ROS-induced lipid peroxidation. Moreover, the fact that 40% of control gonocytes showed lipid peroxidation without affecting their viability, and that lipid peroxidation was not proportional to viability in cells treated with PRDX inhibitors, suggest that gonocytes can tolerate a certain level of lipid peroxidation. The finding that MJ33 treatment increased lipid peroxidation suggests that the PRDX6 iPLA_2_ activity also plays a role in repairing oxidized membranes [27]. 

### 3.4. PRDX Inhibition Blocks RA-Induced Differentiation in PND3 Gonocytes

**Figure 6:**
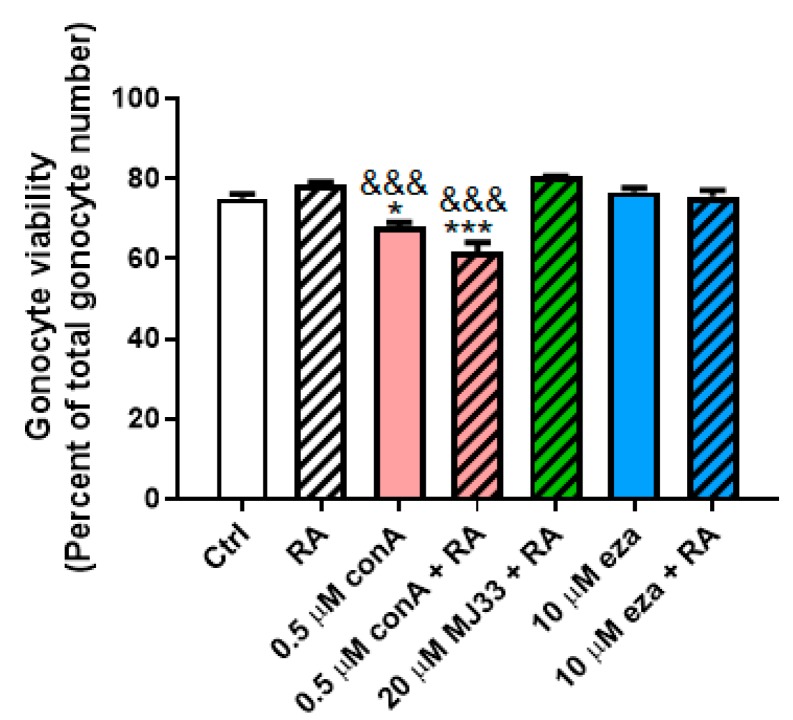
Effects of trans-retinoic acid (RA) and PRDX inhibitors on PND3 gonocyte viability. RA (1 μM) and PRDX inhibitors at the indicated concentrations were added alone or together for two hours on gonocytes. Cell viability was determined by the trypan blue exclusion method. Results represent the means ± SEM from at least 3 experiments. Statistical significance: * *p* ≤ 0.05, *** *p* ≤ 0.001, treatment against control; ^&&&^ *p* ≤ 0.001, treatment against RA alone.

Considering the physiological roles played by ROS in many tissues and cell types, including in spermatozoa, and our finding that inhibiting PRDXs increases endogenous ROS formation in PND3 gonocytes, we examined whether blocking PRDXs could affect RA-induced gonocytes differentiation. First, we determined cell viability in cells treated for two hours with RA alone or together with two different concentrations of PRDX inhibitors, in order to select concentrations of inhibitors that would not overly decrease cell survival. RA at 1 μM did not have an effect on cell survival. Similarly, concentrations of 20 μM MJ33 or 10 μM ezatiostat did not affect cell viability (Figure 6). While 0.5 μM conoidin A decreased cell viability by 9% when used alone, and 17% when added with RA in comparison to control and RA treatments, these was still relatively minor effects. 

**Figure 7:**
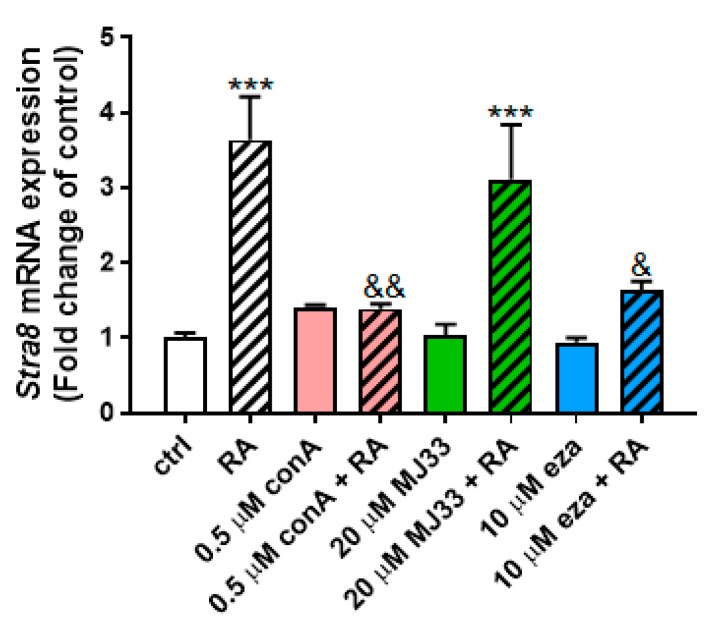
Effects of trans-retinoic acid (RA) and PRDX inhibitors on PND3 gonocyte differentiation. RA (1 μM) and PRDX inhibitors at the indicated concentrations were added alone or together for two hours on gonocytes. *Stra8* mRNA expression was measured by qPCR analysis. Results represent the means ± SEM from three experiments. Statistical significance: *** *p* ≤ 0.001, treatment against control; ^&^ *p* ≤ 0.05, ^&&^ *p* ≤ 0.01, treatment against RA alone.

Next, we measured the mRNA expression of Stra8, previously found to be a good marker for assessing neonatal gonocyte differentiation [15,16,17]. While two hours of treatment with RA significantly increased Stra8 expression by over three-fold, there was no effect of conoidin A, MJ33 or ezatiostat added alone to the cells (Figure 7). However, both conoidin A and ezatiostat significantly repressed RA-induced Stra8 increases, indicating inhibitory effects of PRDX inhibitors on gonocytes differentiation. However, MJ33 did not alter RA effect on Stra8 levels. These data suggest that the oxidative stress resulting from PRDXs inhibition is detrimental to gonocytes differentiation, whereas PRDX6 iPLA activity does not appear to be required for gonocyte differentiation. 

## 4. Discussion

The goal of the present study was to determine whether peroxiredoxins play a role in the survival of neonatal male germ cells, as it is the case in spermatozoa. We hypothesized that neonatal germ cells and spermatozoa would likely require different antioxidant machineries, in view of their considerable molecular, morphological and functional differences, as well as their distinct cellular environments. Indeed, the most abundant antioxidants genes in PND3 gonocytes were *Txn1*, *Prdx1*, *Sod1*, *Prdx2, Prdx6*, and *Prdx5*, whereas a mixed population of adult germ cells expressed *Sod1* at a similar level as these genes, followed by *mGst1*, *Prdx1*, *Prdx6*, *Txn1*, *Prdx2* and *Prdx5* at lower levels. These data indicate that neonatal gonocytes express very high levels of antioxidant genes relative to adult germ cells, although one cannot exclude that some of these genes might be highly expressed in discreet subsets of adult spermatogonia, spermatocytes, or spermatids. The requirement for high levels of antioxidant genes in neonatal gonocytes might reflect the production of ROS during their multiple functions, including DNA methylation, cell proliferation, migration, and differentiation, all processes requiring energy and the generation of ROS [13,14].

Indeed, our finding that the direct inhibition of 2-Cys PRDX with conoidin A and the specific inhibition of GSTP1, enzyme necessary for the re-activation of PRDX6 peroxidase activity, with ezatiostat [28,29,30,31] induce rapid and extensive cell death in PND3 gonocytes implies that high levels of ROS are formed in these cells in physiological conditions. This further suggests that PRDXs play an essential role in maintaining ROS at levels required for physiological functions, but not high enough to induce cell damage. Our results showing that lipid peroxidation is greatly increased by these inhibitors and is associated with increased cell death suggest that lipid peroxides are in part responsible for the adverse effects on gonocytes. In this context, high levels of antioxidant genes, in particular PRDXs, protect neonatal gonocytes from damaging levels of ROS. Moreover, the data clearly show that other antioxidant genes are not capable of rescuing gonocytes from the deleterious effects of ROS accumulation in the absence of PRDXs. In this regard, PRDXs are clearly essential to the maintenance on ROS homeostasis and gonocytes survival, as they are in spermatozoa [4,6].

Recently, it was reported that as human PRXD6, targeted to the yeast mitochondrial matrix, elicited glutathione disulfide (GSSG) formation upon treatment of cells with H_2_O_2_ [32]. Because yeast lack GSTP1, it was suggested that other enzymes may re-activate the peroxidase activity of PRDX6. Although we observed that glutaredoxin 1 (GRX1) and other GSTs were present in gonocytes, the level of their expression was lower than that of GSTP1 (Figure 1; Table S1). This finding and the decrease in gonocyte survival due to the inhibition of GSTP1 by ezatiostat, indicate that the re-activation of PRDX6 peroxidase activity is probably accomplished by GSTP1 and GSH in gonocytes, as described in other mammalian cells [33].

The finding, that conoidin A and ezatiostat both greatly aggravated H_2_O_2_ adverse effects, suggests that 2-Cys PRDXs, likely PRDX1 and 2 which were the most abundant at the protein level, as well as PRDX6 are the peroxidases that actively remove ROS such as H_2_O_2_ from neonatal gonocytes exposed to oxidative stress. Taken together with the fact that Sertoli cells produce H_2_O_2_ [20], these data suggest that the exposure of neonatal gonocytes to ROS produced by Sertoli cells might be part of a physiological process, as it happens in various tissues and cell types [8,34,35,36]. However, as shown by the effects of PRDX inhibitors, ROS levels need to be tightly regulated in order to maintain ROS at non-toxic levels in gonocytes. 

The fact that the PRDX inhibitors conoidin A and ezatiostat both decrease RA-induced gonocyte differentiation suggests that PRDXs are essential to control ROS levels, preventing cell death and allowing some of the cells to undergo gonocyte differentiation. Moreover, the finding that PRDX inhibition promoted high levels of ROS, which prevented gonocyte differentiation without impairing viability, suggests that one way by which the Sertoli cells could control the timing of gonocyte differentiation would be by producing ROS at levels that would prevent or allow gonocyte differentiation. Further studies are necessary to confirm this possibility. Considering recent studies showing that gonocytes are heterogeneous, similarly to spermatogonia [14,37,38], it would be interesting to study whether neonatal testes contain sub-sets of gonocytes with different PRDX activities, corresponding to different functional states. 

One of the main differences we found with our studies on PRDXs in spermatozoa is related to the role of the PRDX6 iPLA_2_ activity. While this activity is critical for the survival and fertilizing ability of spermatozoa by repairing oxidized membranes [10,11,12], our results with MJ33 suggest that the PLA_2_ activity of PRDX6 plays a role in gonocyte survival mainly in the presence of exogenous oxidative stress. Yet, the increased cell death observed after treating gonocytes for 18 h with 50 μM MJ33 suppressing PRDX6 iPLA_2_ activity implies that impairing the repair of oxidized membranes in gonocytes can jeopardize PND3 gonocyte survival over time. We recently reported that exogenous addition of arachidonic acid or lysophosphatidic acid (LPA) prevented cell death in spermatozoa treated with MJ33 [39]. The addition of H_2_O_2_ to cells where PRDX6 iPLA_2_ is inactive and possibly arachidonic acid levels were depleted is sufficient to induce a large increase in cell death in only two hours. Thus, excessive levels of ROS combined with an increased lipid peroxidation and depletion of arachidonic acid and/or LPA appear to aggravate the fate of the cells.

Interestingly, we recently reported that neonatal gonocytes express COX2 and other enzymes of the prostaglandin pathway, and produce prostaglandins (PG) E2 and PGF2a [19]. Moreover, blocking PGE2 and PGF2a synthesis with ibuprofen for 24 h correlated with a partial decrease in RA-induced differentiation in PND3 gonocytes [19]. However, inhibiting the PRDX6 iPLA_2_ activity for 2 h does not seem to affect gonocyte differentiation. Since arachidonic acid is the precursor of prostaglandins, the present data suggest that a short two-hour treatment with MJ33 may not deplete arachidonic acid sufficiently to compromise cell differentiation, or that this process involves other PLA_2_ enzymes expressed in gonocytes [19]. 

## 5. Conclusions

In conclusion, the present study identified PRDX1, 2, 6, and 5 as major antioxidant genes and proteins in neonatal gonocytes, essential for the survival of the cells, especially under conditions of oxidative stress. Moreover, gonocytes were more sensitive to Conoidin A effects than spermatozoa, whereas they were less sensitive to the inhibition of the PLA_2_ activity of PRDX6 than spermatozoa, suggesting a difference in the antioxidant machinery of gonocyte and spermatozoa. Further studies will be needed to examine the possibility that ROS, endogenous or produced by Sertoli cells, at non-toxic levels, might play a role in the regulation of neonatal gonocyte differentiation. 

## Figures and Tables

**Table 1:** Primer sets for qPCR analysis of genes in rat gonocytes. The underlined bases correspond to bases added to the gene sequence by the primer design program to generate better-balanced primers.

Gene	Accession Number	Forward and Reverse Primers
18S rRNA	X01117.1	Cgggtgctcttagctgagtgtcccg ctcgggcctgctttgaacac
Stra8	XM_575429.2	tgcttttgatgtggcgagct gcgctgatgttagacagacgct
Peroxiredoxin 1 (PRDX1)	NM_057114.1	acctgtagctcgactctgctg aacagccgtggctttgaa
PRDX2	NM_017169.1	gactctcagttcacccacctg tattcagtgggcccaagc
PRDX3	NM_022540.1	agaagaacctgcttgacagaca caggggtgtggaatgaaga
PRDX4	NM_053512.2	tgagacactgcgtttggttc tgtttcactaccaggtttccag
PRDX5	NM_053610.1	agtgccgcggtgactatg caaaacacctttcttgtccttga
PRDX6	NM_053576.2	ttgattgctctttcaatagactctg ctgcaccattgtaagcattga

## Supplementary Materials

The following are available online at https://www.mdpi.com/2076-3921/9/1/32/s1, Table S1: Relative gene expression of antioxidant genes expressed in rat PND3 gonocytes and PND8 spermatogonia. The results represent the mean ± SEM of 3 independent RNA samples for each age, each made from cells isolated in multiple animals. There was no significant difference between the two types of germ cells for any of the genes. A few genes presenting a signal intensity below 20 were not included.
