# Supplementary material for: Protective Role of Peroxiredoxins against Reactive Oxygen Species in Neonatal Rat Testicular Gonocytes
Source: Antioxidants (Basel). 2019 Dec 30;9(1):32. doi: 10.3390/antiox9010032 (PMC7022870; doi:10.3390/antiox9010032)
Supplement: Supplementary file 1 [file antioxidants-09-00032-s001.pdf]

Supplementary Table S1. Relative gene expression of antioxidant genes expressed in rat PND3 gonocytes and PND8 spermatogonia. The results represent the mean ± SEM of three independent RNA samples for each age, each made from cells isolated in multiple animals. There was no significant difference between the two types of germ cells for any of the genes. A few genes presenting a signal intensity below 20 were not included.

Gene	Gonocytes			Spermatogonia		
Symbol	mean	±	SEM	mean	±	SEM
Prdx1	2302	±	88	2161	±	63
Prdx2	1178	±	192	1186	±	89
Prdx3	471	±	51	478	±	16
Prdx5	1100	±	87	862	±	67
Prdx6	932	±	71	915	±	61
Sod1	1703	±	110	1860	±	34
Sod2	632	±	146	357	±	48
Sod3	24	±	10	27	±	3
Cat	44	±	6	53	±	4
Gstp1	1161	±	269	655	±	156
Gpx4	1073	±	114	1030	±	77
Gsto1	542	±	80	433	±	47
Mgst1	387	±	29	291	±	32
Gstp2	343	±	103	226	±	51
Gpx1	340	±	37	355	±	17
Glrx1	73	±	20	54	±	9
Gstt2	65	±	22	69	±	4
Hagh	61	±	2	71	±	8
Gpx2	49	±	5	16	±	2
Gstt1	46	±	11	32	±	6
Gstk1	43	±	3	56	±	3
Gstm2	26	±	6	34	±	4
Gpx3	23	±	1	21	±	3
Gss	93	±	10	81	±	8
Gsr	90	±	7	77	±	6
Txn1	4198	±	312	3762	±	305
Txnl1	432		28	588		73
Txnrd1	348	±	38	229	±	27
Txn2	83	±	12	86	±	5
Txnrd2	58	±	1	65	±	4
Keap1	54	±	9	49	±	3
Nrf2 (Nfe2l2)	407	±	52	379	±	40
